# ID 161 - Análise de Custos e Retorno de Escala da Ampliação da Oferta de Profilaxia Pré-Exposição (PrEP) para Populações-Chave de 15 a 17 Anos em Duas Cidades Brasileiras

**DOI:** 10.5327/2237-9622.2025.v34s1.161

**Published:** 2025-11-25

**Authors:** Joilson Nascimento Paim, Laio Magno, Patricia Coelho de Soarez, Natacha Cerchiari, Ines Dourado, Alexandre Grangeiro, Andreia Costa Santos

**Keywords:** análise de custos, custo financeiro, custo econômico, análise de ampliação, retorno de escala, função Cobb-Douglas, HIV/aids, homens que fazem sexo com homens, mulheres transgênero

## Abstract

**Introdução:**

No Brasil, a incidência de infecções por HIV está aumentando, especialmente entre homens que fazem sexo com homens (HSH), travestis e mulheres trans (TrMT). A profilaxia pré-exposição (PrEP) é uma estratégia eficaz de prevenção, oferecida gratuitamente para pessoas a partir de 15 anos no Sistema Único de Saúde (SUS), porém sem uma estratégia consistente de criação de demanda (ECD) para apoiar as metas dos Objetivos de Desenvolvimento Sustentável, adotados pela Organização das Nações Unidas em 2015. Os objetivos deste estudo são avaliar: (1) o custo total e o custo médio total da oferta de PrEP, incluindo a ECD, direcionada a adolescentes HSH e TrMT com idades entre 15 e 17 anos; e (2) os benefícios da ampliação da cobertura de PrEP para essa população-alvo no SUS.

**Método:**

Estimamos o custo total e o custo médio total, e os ganhos de escala para a expansão da oferta de PrEP no SUS usando funções de Cobb-Douglas.

**Resultados:**

O custo médio total por oferta de PrEP foi estimado em USD 320 em Salvador e USD 253 em São Paulo. Ganhos de escala foram observados em ambos os cenários de estudo e também em âmbito nacional para o SUS.

**Conclusão:**

Nossas estimativas indicam que os investimentos na expansão da oferta de PrEP para adolescentes de 15 a 17 anos provavelmente reduzirão os custos médios totais para o SUS. No entanto, seria necessária uma análise de custo-efetividade para avaliar se esses investimentos na expansão da oferta de PrEP maximizariam os benefícios na redução da incidência de HIV/aids entre a população-alvo, em comparação com as práticas atuais do SUS.

